# Recent advances in mineralocorticoid receptor antagonists for heart failure with preserved ejection fraction: focus on finerenone in the era of sodium-glucose cotransporter-2 inhibitors and glucagon-like peptide-1 receptor agonists

**DOI:** 10.3389/fphar.2025.1725782

**Published:** 2025-12-16

**Authors:** Tian-Yu Wang, Lei Zhang, Huan-Yi Wang, Fen-Fen Jiang

**Affiliations:** Department of Geriatrics, The Second Affiliated Hospital of Jiaxing University, Jiaxing, China

**Keywords:** heart failure with preserved ejection fraction (HFpEF), chronic kidney disease (CKD), mineralocorticoid receptor antagonists (MRAs), finerenone, sodium-glucose cotransporter-2 inhibitors (SGLT2is), glucagon-like peptide-1 receptor agonists (GLP-1 RAs)

## Abstract

Heart failure with preserved ejection fraction (HFpEF) is a clinically diverse disease characterized by intricate pathophysiological pathways, for which effective treatment options remain limited. Aberrant activation of the mineralocorticoid receptor (MR) significantly contributes to the development and progression of HFpEF. Finerenone, an innovative non-steroidal mineralocorticoid receptor antagonists (MRAs), has enhanced MR selectivity, more potent anti-fibrotic and anti-inflammatory effects, and improved safety relative to conventional steroidal MRAs like spironolactone and eplerenone. Therefore, future large-scale phase III head-to-head randomized controlled trials comparing finerenone and spironolactone in the HFpEF population, with cardiovascular outcomes as the primary endpoint, will be crucial. In preclinical models, finerenone has demonstrated improvement in multiple pathophysiological parameters of HFpEF. The FIDELIO-DKD and FIGARO-DKD trials in individuals with chronic kidney disease (CKD) and type 2 diabetes mellitus (T2DM) initially demonstrated finerenone’s cardiorenal advantages, including substantial decreases in cardiovascular events and the risk of renal function decline. The FINEARTS-HF trial has expanded this data to patients with HFmrEF/HFpEF, showing a significant decrease in the incidence of total worsening HF events and mortality from cardiovascular causes. Additionally, the potential for combining finerenone with sodium-glucose cotransporter-2 inhibitors (SGLT2is) or glucagon-like peptide-1 receptor agonists (GLP-1 RAs) is gaining attention. Current trials, including REDEFINE-HF, CONFIRMATION-HF, and FINALITY-HF, are examining its effectiveness across various HF phenotypes. These research will elucidate finerenone’s function in the management of cardiometabolic disorders. This review focuses on the clinical evidence of finerenone in patients with HFpEF and concomitant CKD, along with its potential cardiorenal protective mechanisms. It aims to provide new evidence-based medical evidence and theoretical support for the clinical management of HFpEF patients.

## Introduction

Heart failure (HF) is a multifaceted clinical illness defined by anatomical or functional impairments of the heart, leading to elevated intracardiac pressures and inadequate cardiac output during rest or exertion (McDonagh et al., 2023). HF is a significant contributor to cardiovascular morbidity and mortality, emerging as a critical global public health challenge that impacts over 64 million individuals globally and places a substantial strain on society and healthcare systems (Savarese et al., 2023). Based on left ventricular ejection fraction (LVEF), HF is classified into three types: HF with reduced ejection fraction (HFrEF), HF with mildly reduced ejection fraction (HFmrEF), and HF with preserved ejection fraction (HFpEF) (Heidenreich et al., 2022). HFpEF represents a clinically heterogeneous syndrome with complex pathophysiological mechanisms, commonly characterized by impaired LV diastolic function and elevated LV filling pressures (Borlaug et al., 2023). Among all adults hospitalized for HF, HFpEF constitutes up to 50% of instances. Compared to HFrEF patients, HFpEF patients exhibit higher prevalence of obesity, type 2 diabetes mellitus (T2DM), hypertension, atrial fibrillation, chronic kidney disease (CKD), and other non-cardiovascular comorbidities (Campbell et al., 2024).

Among these, CKD and HFpEF have comparable pathophysiological processes and a reciprocal interaction that contributes to the development and course of both diseases. About 55% of individuals with HFpEF have at least CKD stage 3a, while 20% of patients with CKD have new-onset HF (Tuttle et al., 2024). Patients with CKD often exhibit more severe symptoms and greater impairment in cardiac structural and functional parameters compared to those without CKD. Moreover, the severity of CKD correlates with adverse outcomes, including cardiovascular mortality and HF hospitalization (Bonacchi et al., 2024). This synergistic pathophysiology renders the management of HFpEF in CKD patients particularly challenging. Over the past 2 decades, treatment approaches for HFrEF have evolved, with multiple medications demonstrating mortality-reducing effects. However, effective therapies for HFpEF remain elusive (Omote et al., 2022). Prior to the landmark EMPEROR-Preserved study results, managing HFpEF remained challenging. Based on EMPOROR-Preserved findings, the 2022 American HF guidelines classified sodium-glucose cotransporter-2 inhibitors (SGLT2is) as a Class IIa recommendation for HFpEF treatment (Anker et al., 2021). In randomized controlled trials (RCTs) for HFpEF, drugs proven effective for HFrEF, including renin-angiotensin system inhibitors (RASIs), beta-blockers, mineralocorticoid receptor antagonists (MRAs), and nitric oxide-cyclic guanosine monophosphate-protein kinase G pathway stimulators, did not achieve primary outcome. SGLT2is are the only agents proven in RCTs to improve cardiovascular outcome in HFpEF patients (Cohen et al., 2023). Furthermore, results from the phase III STEP-HFpEF and STEP-HFpEF DM trials demonstrated that weekly subcutaneous injections of 2.4 mg semaglutide significantly reduced HF-related symptoms and physical limitations while improving exercise capacity in obese HFpEF patients, suggesting potential expansion of cardiovascular indications for glucagon-like peptide-1 receptor agonists (GLP-1 RAs) (Kosiborod et al., 2023; Kosiborod et al., 2024). Numerous recent clinical investigations demonstrate that finerenone also holds considerable promise for treating HFpEF. Recent data from the FIDELIO-DKD and FIGARO-DKD trials indicate that finerenone reduces the risk of CKD progression and HF hospitalization in participants with CKD and T2DM compared to placebo (Bakris et al., 2020; Pitt et al., 2021). This drug has now been approved by the U.S. Food and Drug Administration for treating diabetic kidney disease (DKD) and adult HF patients with LVEF ≥ 40% (Beghini et al., 2025). This development has shered in new hope and transformative change for the treatment of HFmrEF/HFpEF. Finerenone stands as the first MRA to demonstrably improve clinical outcome in HF patients with LVEF ≥ 40%.

This review summarizes the existing clinical evidence for finerenone in patients with HF, emphasizing its cardiovascular advantages in individuals with HFpEF. Furthermore, we delineate the bidirectional interaction between HFpEF and CKD and investigate the cardiorenal protective mechanisms of finerenone in the management of HFpEF patients with concurrent CKD. The need for specific finerenone clinical studies in HFpEF patients is further highlighted by these clinical results and molecular insights.

## Overview of MR and its antagonists

The mineralocorticoid receptor (MR) belongs to the steroid receptor superfamily. MR is expressed in nearly all nephron segments and cardiomyocytes, including the distal convoluted tubule, collecting duct, vascular endothelial cells (ECs), podocytes, mesangial cells of the kidney, as well as cardiomyocytes, vascular smooth muscle cells (VSMCs), fibroblasts, and immune cells of the heart (Bauersachs and López-Andrés, 2022). For several endogenous hormones, including as progesterone, cortisol, and aldosterone, the MR shows comparable affinity (Ibarrola and Jaffe, 2024). Overexpression of the MR can promote proinflammatory factors such as tumor necrosis factor-alpha (TNF-α), interleukin-6 (IL-6), monocyte chemoattractant protein-1 (MCP-1), and pro-fibrotic factors like transforming growth factor-beta (TGF-β) and collagen I/III, leading to myocardial hypertrophy, interstitial fibrosis, and vascular endothelial dysfunction. This ultimately facilitates the advancement of HF and CKD (Savarese et al., 2024). In response to the abnormal enhancement of MR activity, MRAs were developed. Traditional steroid MRAs include spironolactone and eplerenone. Spironolactone, a first-generation MRA, possesses potent MR antagonistic activity and is widely used in HFrEF and resistant hypertension (Camafort et al., 2024). The TOPCAT trial partially suggested it may offer some benefit in HFpEF patients (Pitt et al., 2014). However, spironolactone’s non-selective action on sex hormone receptors predisposes patients to adverse consequences associated with sex hormones, including menstruation abnormalities and gynecomastia (Ferreira et al., 2024). As a second-generation MRA, eplerenone exhibits higher MR selectivity, markedly reducing sex hormone-related adverse effects. It has been shown to enhance survival rates in HFrEF patients and post-myocardial infarction HF (Ambrosy and Gheorghiade, 2011). Nevertheless, long-term use of both agents carries risks of renal deterioration and hyperkalemia, particularly pronounced in HFpEF patients with concomitant CKD, limiting their widespread application. Finerenone, a dihydropyridine-based compound, exhibits higher MR affinity and tissue selectivity compared to traditional MRAs. It also crosses the blood-brain barrier less readily, reducing central nervous system side effects (Heinig and Eissing, 2023). Finerenone demonstrates comparable distribution in cardiac and renal organs, in contrast to spironolactone and eplerenone, which predominantly concentrate in the kidneys. This distribution pattern may explain finerenone’s significant cardioprotective effects (González-Juanatey et al., 2023).

In summary, the MR and its associated signaling pathways are pivotal in the pathophysiological advancement of cardio-renal disorders. MR-targeted therapy is gradually entering an era of greater precision and safety, offering new therapeutic opportunities for patients with HFpEF. Further exploration is warranted into the potential combination of MRAs with other cardiorenal metabolic agents, such as SGLT2is or GLP-1 RAs.

## The interplay between HF and CKD

HFpEF and CKD frequently coexist clinically, sharing complex and intimate pathophysiological connections that constitute a classic heart-kidney interaction. In HFpEF patients, the prevalence of CKD reaches 40%–70%; conversely, CKD patients exhibit significantly increased risk of developing HF, particularly HFpEF (Patel et al., 2023). This comorbidity not only exacerbates clinical symptoms and increases hospitalization and mortality rates but also poses significant challenges for therapeutic management. Deepening our understanding of the interaction mechanisms between HFpEF and CKD holds substantial clinical significance for exploring novel intervention targets and optimizing integrated treatment strategies.

The relationship between different types of HF and CKD varies slightly. According to several studies, HFpEF has a substantially greater prevalence of CKD than HFmrEF and HFrEF (Szlagor et al., 2023). In recent pivotal trials such as DELIVER and FINEARTS-HF, approximately fifty percent of recruited patients had an eGFR of less than 60 mL/min/1.73 m^2^ (Mc Causland et al., 2025; Solomon et al., 2022). A prospective US study involving 299 HFpEF patients employed conventional 2-D and speckle-tracking echocardiography to delineate cardiac anatomical and functional characteristics in HFpEF patients with and without CKD. Results indicated that patients with CKD were older, had higher B-type natriuretic peptide (BNP) levels, and were more likely to have T2DM and hypertension, with worse New York Heart Association (NYHA) functional class. Even after multivariate adjustment, LV longitudinal strain, right ventricular free wall strain, and left atrial strain parameters remained significantly correlated with CKD (Unger et al., 2016). A meta-analysis incorporating 57 studies and 1,076,104 patients examined the impact of CKD on mortality in HF patients, revealing that moderate-to-severe renal impairment is an independent predictor of mortality (Damman et al., 2014). HFpEF and CKD exhibit bidirectional associations, with significant overlap in disease drivers and progression risk factors such as hypertension, T2DM, obesity, dyslipidemia, and smoking. The prevalence of these conditions increases with age (Namoos et al., 2024). However, beyond these shared susceptibility factors, genuine mechanistic pathways also exist (Jankowski et al., 2021). CKD and HFpEF have similar pathophysiological mechanisms, including systemic inflammation, oxidative stress, elevated neurohormone levels, venous congestion, and overactivation of the renin-angiotensin-aldosterone system (RAAS). Each of these elements has a part in the onset and advancement of CKD and HFpEF (Zannad and Rossignol, 2018). Current understanding emphasizes the pivotal role of systemic inflammation in the pathophysiology of HFpEF and CKD. This chronic low-grade inflammation leads to increased reactive oxygen species (ROS) production, reduced nitric oxide synthesis, and coronary microvascular endothelial dysfunction, subsequently triggering myocardial hypertrophy and interstitial fibrosis (Kingma et al., 2017; Ter Maaten et al., 2016). Mitochondrial dysfunction, specific pro-fibrotic factors, and RAAS overactivation also contribute to this process (Mendez-Barbero et al., 2023). Notably, these processes are interconnected and mutually amplifying. This association perhaps attributable to reduced eGFR, a critical marker of CKD that is also significantly correlated with increased all-cause and cardiovascular mortality in HFpEF patients (Kidney Disease: Improving Global Outcomes KDIGO CKD Work Group, 2024; House et al., 2019). Moreover, HFpEF patients commonly exhibit chronotropic incompetence, which is independently correlated with diminished eGFR (Klein et al., 2015). Moreover, congestion represents an often underestimated factor in the pathophysiology of both HFpEF and CKD. Elevated central venous pressure in HFpEF patients increases renal interstitial pressure, compressing the glomeruli, tubules, and renal veins within the encapsulated kidney, giving rise to the “kidney tamponade hypothesis” (Boorsma et al., 2022). This effect is reciprocal, in patients with advanced CKD, poor salt and water processing raises preload, further promoting cardiac remodeling. Novel hemodynamic parameters may aid in elucidating additional pathophysiological mechanisms.

Building upon the in-depth elucidation of the cardiorenal cross damage mechanism, novel combined intervention strategies targeting the cardiorenal axis have gradually emerged. Among these, MRAs have garnered particular attention. As a key receptor target in the cardiorenal axis, aberrant activation of the MR significantly contributes to cardiac remodeling but also mediates renal interstitial fibrosis and the onset of proteinuria (Kintscher et al., 2022). The next-generation nonsteroidal MRA finerenone, with its highly selective MR antagonism and potent anti-inflammatory and anti-fibrotic properties, shows promising application prospects in patients with HFpEF complicated by CKD. Additionally, novel metabolic agents such as SGLT2is and GLP-1 RAs improve cardiorenal axis function through multiple mechanisms, offering new opportunities for synergistic therapy. Future multi-targeted combined interventions for patients with HFpEF and CKD will become a key development direction for precision treatment of cardio-renal comorbidity.

## Clinical evidence of finerenone in HFpEF

In recent years, as research into the pathogenesis of HFpEF has deepened, clinical studies have increasingly focused on drugs that simultaneously exhibit anti-inflammatory, anti-fibrotic, and dual cardiorenal protective effects. As a new-generation MRA, finerenone’s high selectivity, few side effects, and complex mode of action have made it a viable therapeutic choice for HFpEF. Although initially studied primarily for its renal protective effects in patients with CKD and T2DM, its cardiovascular benefits have been confirmed in large-scale RCTs, sparking widespread interest in its efficacy for HFpEF patients (Table 1).

**TABLE 1:** Clinical trials overview of the effects of MRAs in patients with HFmrEF/HFpEF or patients with CKD and T2DM.

Trial	Trial design	MRAs	Trial population	Baseline HF	Median follow-up	HF hospitalization	Additional results	References
Mottram et al.	Randomized, double-blind, placebo-controlled trial	Spironolactone 25 mg once a day vs. Placebo	30 medically treated hypertensive patients with exertional dyspnea, EF > 50%, and diastolic dysfunction and without ischemia	100%	6 months	NA	Spironolactone significantly increased the long-axis strain rate, peak systolic strain, and cyclic variation of integrated backscatter; improvements in strain were not significantly associated with changes in BP during treatment	Mottram et al. (2004)
RAAM-PEF	Randomized, double-blind, placebo-controlled trial	Eplerenone 50 mg once a day vs. Placebo	44 patients with HFpEF	100%	6 months	NA	Eplerenone was associated with a significant reduction in serum markers of collagen turnover (procollagen type I N-terminal propeptide and carboxy-terminal telopeptide of type I collagen) and improvement in echocardiographic measures of diastolic function E/E'	Deswal et al. (2011)
Kampourides et al.	Open-label, randomized, parallel-group, nonplacebo-controlled trial	Eplerenone 25 mg once a day vs. No eplerenone	303 post-acute myocardial infarction patients with a LVEF ≥ 40%	NA	24 months	NA	In the overall study cohort, use of eplerenone did not improve outcomes. However, among patients with low baseline MMP-9 levels, eplerenone was associated with a significant prognostic benefit	Kampourides et al. (2012)
ALDO-DHF	Multicentre, randomized, double-blind, placebo-controlled trial	Spironolactone 25 mg once a day vs. Placebo	422 patients with chronic HF (NYHA class II-III) with LVEF ≥ 50% and diastolic dysfunction	100%	12 months	NA	Spironolactone improved LV diastolic function in patients with HFpEF, but did not affect peak exercise capacity, symptoms, or quality of life	Edelmann et al. (2013)
Kurrelmeyer et al.	Open-label, randomized, double-blind, placebo-controlled trial	Spironolactone 25 mg once a day vs. Placebo	48 elderly women with HFpEF (NYHA class II-III) with LVEF ≥ 50% and diastolic dysfunction	100%	6 months	0 vs. 0 (no HF hospitalization)	Spironolactone therapy effectively improved diastolic function by significantly increasing the early diastolic tissue Doppler velocity of the lateral mitral annulus (lateral e') and significantly decreasing the ratio of mitral peak E velocity to lateral e' (lateral E/e'). Spironolactone also reduced myocardial fibrosis by lowering levels of type III procollagen	Kurrelmeyer et al. (2014)
TOPCAT	Multicenter, randomized, double-blinded, placebo-controlled, phase 3 trial	Spironolactone 15 mg (titrated up to 30 or 45 mg) once a day vs. Placebo	3445 patients with symptomatic HF and a LVEF of 45% or more	100%	3.3 years	12.0% vs. 14.2%; HR 0.83 (0.69–0.99); P = 0.04	In patients with HFpEF, treatment with spironolactone did not significantly reduce the incidence of the primary composite outcome of death from cardiovascular causes, aborted cardiac arrest, or hospitalization for HF.	Pitt et al. (2014)
Kosmala et al.	Randomized, double-blind, parallel-group, placebo-controlled trial	Spironolactone 25 mg once a day vs. Placebo	150 patients with exertional dyspnea (NYHA class II-III, LVEF>50%, diastolic dysfunction, and exertional E/e'>13)	100%	6 months	NA	In patients with HFpEF and abnormal diastolic response to exertion, improvement in exercise E/e' mediates the beneficial effect of spironolactone on exercise capacity	Kosmala et al. (2016)
Upadhya et al.	Randomized, double-blind, placebo-controlled trial	Spironolactone 25 mg once a day vs. Placebo	80 stable compensated HFpEF patients with controlled BP	100%	9 months	NA	In stable, compensated older HFpEF patients, 9 months of spironolactone 25 mg/day was well-tolerated and reduced BP, but did not improve exercise capacity, quality-of-life, LV mass or arterial stiffness	Upadhya et al. (2017)
FIDELIO-DKD	Multicenter, randomized, double-blind, placebo-controlled, parallel-group, event-driven phase 3 trial	Finerenone 10 or 20 mg once a day vs. Placebo	5,734 patients with CKD and T2DM	NA	2.6 years	4.9% vs. 5.7%; HR 0.86 (0.68–1.08)	In patients with CKD and T2DM, treatment with finerenone resulted in lower risks of CKD progression and cardiovascular events than placebo	Bakris et al. (2020)
FIGARO-DKD	Multicenter, randomized, double-blind, placebo-controlled, parallel-group, event-driven phase 3 trial	Finerenone 10 or 20 mg once a day vs. Placebo	7,437 patients with CKD and T2DM	NA	3.4 years	3.2% vs. 4.4%; HR 0.71 (0.56–0.90)	Finerenone therapy significantly reduced the risk of the primary composite outcome, driven mainly by a marked decrease in HF hospitalization. Therefore, finerenone improved cardiovascular outcomes in patients with T2DM and mild-to-moderate CKD.	Pitt et al. (2021)
FINEARTS-HF	Multicenter, randomized, double-blind, placebo-controlled, parallel-group phase 3 trial	Finerenone (at a maximum dose of 20 mg or 40 mg depending on the baseline eGFR) once a day vs. Placebo	6,001 patients with symptomatic HF, LVEF ≥ 40%, structural heart disease, and elevated natriuretic peptides	100%	32 months	Total worsening HF events (unplanned hospitalization/urgent visit): HR 0.82 (0.71–0.94); P = 0.006	In patients with HFmrEF or HFpEF, finerenone resulted in a significantly lower rate of a composite of total worsening HF events and death from cardiovascular causes than placebo	Solomon et al. (2024)

*Abbreviations*: BP, blood pressure; CKD, chronic kidney disease; eGFR, estimated glomerular filtration rate; HF, heart failure; HFmrEF, heart failure with mildly reduced ejection fraction; HFpEF, heart failure with preserved ejection fraction; HR, hazard ratio; LVEF, left ventricular ejection fraction; MMP-9, matrix metalloproteinase-9; MRAs, mineralocorticoid receptor antagonists; NA, not available; NYHA, new york heart association; P, probability; T2DM, type 2 diabetes mellitus; Vs., versus.

In the FIDELIO-DKD trial, finerenone dramatically decreased the risk of renal deterioration and cardiovascular events in patients with CKD and T2DM. The FIDELIO-DKD trial enrolled 5,734 participants with moderate-to-severe CKD and T2DM. Patients were randomized 1:1 to receive finerenone once daily or placebo in addition to conventional treatment. Conventional treatment included glucose-lowering therapy and the maximum tolerated dose of RASis, such as angiotensin-converting enzyme inhibitors (ACEIs) or angiotensin II receptor blockers (ARBs). The primary composite outcome comprised kidney failure, sustained decrease of ≥ 40% in eGFR from baseline, or death from renal causes. The key secondary composite outcome comprised death from cardiovascular causes, nonfatal myocardial infarction, nonfatal stroke, or hospitalization for HF. The findings showed a noteworthy 18% decrease in the risk of the primary composite outcome in the finerenone group compared with placebo (21.1% vs. 17.8%; hazard ratio [HR] = 0.82; 95% confidence interval [CI] 0.73–0.93; P = 0.001), and a significant 14% reduction in the key secondary composite outcome. Additionally, urinary albumin-to-creatinine ratio (UACR) levels were greatly decreased in the finerenone group. Compared to placebo, the risk of hyperkalemia was marginally increased in the finerenone group, however, merely 2.3% of patients ceased finerenone administration due to hyperkalemia, and no fatalities were reported. Following the publication of FIDELIO-DKD results, finerenone became the first MRA globally demonstrated by RCTs to provide cardiorenal benefits in DKD patients. The FIDELIO-DKD study provided preliminary evidence for possible cardiovascular benefits of finerenone in HFpEF patients (Bakris et al., 2020). Subsequently, the FIGARO-DKD study further strengthened this evidence base. FIGARO-DKD trial enrolled 7,437 participants with mild to moderate CKD and T2DM. Patients were randomized 1:1 to receive either finerenone once daily or placebo on top of standard therapy. The primary composite outcome comprised death from cardiovascular causes, nonfatal myocardial infarction, nonfatal stroke, or hospitalization for HF. Results demonstrated a significant 13% reduction in the risk of the primary composite outcome in the finerenone group compared with placebo (14.2% vs. 12.4%; HR = 0.87; 95% CI 0.76–0.98; P = 0.03). The primary driver of this reduction was a significant 29% decrease in hospitalization for HF. Subgroup analyses demonstrated consistent cardiovascular advantages with finerenone regardless of baseline UACR or eGFR levels. Secondary analyses of the FIGARO-DKD study further revealed that finerenone significantly decreased the risk of new-onset HF by 32% in patients without prior HF history. Compared to the placebo group, the finerenone group experienced a greater incidence of hyperkalemia (5.3% vs. 10.8%), but the proportion requiring discontinuation of study medication was lower (0.4% vs. 1.2%) (Pitt et al., 2021). Extending from the FIDELIO-DKD trial in patients with moderate-to-severe CKD and T2DM to the FIGARO-DKD study in patients with mild-to-moderate CKD, the highly consistent outcomes from both trials demonstrate that finerenone can both delay renal decline and reduce the risk of cardiovascular events in patients with T2DM and CKD of varying severity. These complementary studies collectively establish a robust evidence-based foundation for finerenone in this field. The TOPCAT study trial evaluated the efficacy and safety of spironolactone in HFpEF patients. A total of 3,445 patients with LVEF ≥ 45% and a history of HF hospitalization or elevated BNP were enrolled and randomly assigned to receive spironolactone or placebo. The primary composite outcome comprised death from cardiovascular causes, hospitalization for HF, and aborted cardiac arrest. Unfortunately, spironolactone did not significantly reduce the risk of the primary composite outcome (20.4% vs. 18.6%; HR = 0.89; 95% CI 0.77–1.04; P = 0.14). However, a significant benefit was observed in the Americas subgroup, including the United States and Brazil, suggesting regional differences may influence outcome (Pitt et al., 2014). While the TOPCAT study revealed the potential role of MRAs in HFpEF, it also highlighted the heterogeneity of HFpEF and the critical importance of study design, providing valuable reference for subsequent research design. Compared to spironolactone, finerenone exhibits greater MR selectivity, a reduced half-life and a more even distribution in cardiac and renal tissues. It significantly reduces the risk of hyperkalemia and sex hormone-related adverse events, while markedly improving clinical tolerability and compliance (Kintscher, 2023). A study conducted by the Cleveland Clinic in the United States analyzed data from the global health research network TriNetX. Using propensity score matching, two matched cohorts were created, each comprising 142 HFpEF patients in the finerenone group and the spirolactone group. The primary endpoint was a composite of acute myocardial infarction, acute HF, and stroke. Results showed that compared with the spirolactone group, the finerenone group demonstrated a significant reduction in MACE risk. At 30 days, the risk decreased by 62% (26.06% vs. 11.0%; HR = 0.38; 95% CI 0.21–0.68; P = 0.001), and a 32% reduction at 1 year (45.07% vs. 33.80%; HR = 0.68; 95% CI 0.47–0.99; P = 0.043). The finerenone group demonstrated a significant reduction in all-cause mortality, with risk decrease of 75% at 30 days (7.04% vs. 0%; HR = 0.25; 95% CI 0.12–0.71; P = 0.026), 76% at 1 year (14.79% vs. 7.04%; HR = 0.24; 95% CI 0.09–0.64; P = 0.002), and 61% at 5 years (30.28% vs. 9.86%; HR = 0.39; 95% CI 0.21–0.71; P = 0.001), respectively. The finerenone group demonstrated a significant reduction in acute HF risk, with a 70% decrease at 30 days (11.27% vs. 7.04%; HR = 0.30; 95% CI 0.11–0.83; P = 0.013), and a 47% reduction at 1 year (20.42% vs. 11.27%; HR = 0.53; 95% CI 0.29–0.97; P = 0.037). This study supports finerenone’s superiority over spironolactone in improving cardiovascular outcomes and survival rates among patients with HFpEF (Issa et al., 2025). However, as observational studies, even with propensity score matching to reduce selection bias, they cannot fully replace the evidence strength of head-to-head RCTs. Another short-term, phase II comparative trial, the ARTS study and its related analyses, demonstrated that finerenone and spirolactone were comparable in terms of biomarkers and tolerability during short-term follow-up. The ARTS study evaluated multiple-dose regimens of finerenone in patients with HF and CKD, incorporating comparative data against spirolactone (short-term follow-up 29 ± 2 days). Results demonstrated that finerenone reduced biomarkers such as N-terminal pro B-type natriuretic peptide (NT-proBNP) and BNP to a degree comparable to spirolactone, while exhibiting lower rates of hyperkalemia and worsening renal function events (Pitt et al., 2013). This represents one of the few direct head-to-head comparisons currently available in the literature, however, small sample sizes and short-term data cannot substitute for large-scale outcome trials. Therefore, future large-scale phase III head-to-head RCTs comparing finerenone and spironolactone in the HFpEF population, with cardiovascular outcomes as the primary endpoint, will be crucial. To clarify the specific efficacy and safety of finerenone in HFpEF patients, the FINEARTS-HF study was initiated. The FINEARTS-HF trial included 6,001 symptomatic HF patients with LVEF ≥ 40%, who were randomized in a 1:1 ratio to receive either finerenone or a placebo, alongside usual care. The primary composite outcome comprised total worsening HF events and death from cardiovascular causes. Secondary outcomes included change from baseline in the Kansas City Cardiomyopathy Questionnaire (KCCQ) total symptom score at 6, 9, and 12 months; improvement in NYHA functional class at 12 months; kidney composite outcome; death from any cause; and first worsening HF event or death from cardiovascular causes. The study indicated that, in comparison to the placebo group, patients receiving finerenone exhibited a markedly diminished risk of the primary composite outcome by 16% (rate ratio [RR] = 0.84; 95% CI 0.74–0.95; P = 0.007). In a sensitivity analysis of the composite endpoint of first worsening HF event or death from cardiovascular causes, the finerenone group exhibited a markedly reduced risk compared to the placebo group. Subgroup analysis further indicated that the greater the severity of HF symptoms, the greater the benefit of initiating finerenone therapy earlier. For quality-of-life outcomes, the least-squares mean (±SE) change from baseline in KCCQ total symptom score, based on data at 6, 9, and 12 months, was used to estimate the overall treatment effect. The finerenone group showed an improvement of 8.0 ± 0.3 points, while the placebo group improved by 6.4 ± 0.3 points. Furthermore, multiple subgroup analyses from the FINEARTS-HF study exhibited that finerenone’s efficacy in patients with HF and LVEF ≥ 40% remained consistent across different baseline characteristics, including baseline LVEF, NT-proBNP, renal function, blood glucose levels, history of atrial fibrillation, and use of SGLT2is (Solomon et al., 2024). As a groundbreaking study in the field of HF, FINEARTS-HF broke through the treatment impasse for patients with LVEF ≥ 40%, establishing finerenone as the inaugural MRA to exhibit conclusive cardiovascular advantages in HFpEF patients. The FINE-HEART study is a pre-specified individual patient data meta-analysis based on data from three pivotal Phase III RCTs (FIDELIO-DKD, FIGARO-DKD, and FINEARTS-HF), encompassing 18,991 patients at high risk for cardiovascular, renal, and metabolic complications. It aims to systematically evaluate the cardiovascular and renal protective effects of finerenone across different clinical phenotypes. The studied population exhibited a mean age of 67 ± 10 years, with approximately one-third being female. Twelve point one percent of participants had all cardiovascular-kidney-metabolic (CKM) syndrome (HF, CKD, and diabetes). Regarding the primary outcome, the trial failed to demonstrate that finerenone substantially diminished cardiovascular mortality risk, potentially due to differing definitions of cardiovascular death and classifications of unexplained death across the trials. Nevertheless, the finerenone group demonstrated favorable results for secondary outcomes. Compared with placebo, finerenone significantly reduced the risk of hospitalization for HF by 17%; a 20% decrease in the risk of kidney composite outcomes; and a 9% decrease in the risk of death from any cause (Vaduganathan et al., 2024). The FINE-HEART research results unequivocally validate the extensive therapeutic advantages of finerenone in individuals with CKM syndrome, providing high-level evidence-based support for its widespread application in complex populations such as those with HFpEF and concomitant DKD. It also highlights the complexity and challenges in the therapeutic landscape of CKM syndrome. In addition to the aforementioned trials, real-world results also substantiate the utilization of finerenone in HFpEF patients. The FINE registry study was a multicenter observational real-world study from Spain, with a particular focus on those with HFmrEF/HFpEF. Results demonstrated consistent effects in reducing worsening HF events and improving cardiac function (Alonso Salinas et al., 2025). Another observational study from Europe and the United States analyzed real-world HFmrEF/HFpEF patient cohorts, revealing that finerenone significantly reduced the risk of worsening HF events and death from cardiovascular causes across all baseline risk levels (McDowell et al., 2025). These preliminary data indicate that finerenone not only demonstrates efficacy in RCTs but also possesses feasibility and advantages in routine clinical practice.

The synergistic effects of finerenone with SGLT2is or GLP-1 RAs also warrant attention. However, clinical evidence supporting synergistic effects when finerenone is combined with SGLT2is or GLP-1 RAs remains limited. Evidence from recent clinical trials demonstrating the effectiveness and safety of combination therapy is restricted to pre-specified subgroup analyses stratified by baseline use of SGLT2is or GLP-1 RAs. In the FIDELITY study, subgroup analyses demonstrated that concomitant SGLT2is use did not alter finerenone’s effects on cardiovascular or renal outcome. This conclusion was further supported by a subgroup analysis in the FINEARTS-HF study. Another subgroup analysis in the FIDELITY study found no substantial disparity in the therapeutic efficacy of finerenone on cardiovascular and renal outcome between participants with and without baseline use of GLP-1 RAs (Agarwal et al., 2022). These preliminary statistics provided the rationale for designing the CONFIDENCE trial. The CONFIDENCE study emerged as the world’s first multicenter, randomized, controlled phase 2 clinical trial exploring the simultaneous initiation of finerenone and SGLT2is for treating CKD with T2DM. The study enrolled 818 patients with CKD and T2DM from 14 countries. Participants were randomly allocated in a 1:1:1 ratio to receive finerenone monotherapy, empagliflozin monotherapy, or combination therapy, all on top of maximum tolerated doses of RASis. The primary outcome was the alteration in UACR at 180 days compared to baseline. The findings indicated that combined therapy achieved a 52% reduction in UACR from baseline by day 180. This reduction represented an additional 29% decrease compared to finerenone monotherapy and an additional 32% decrease compared to empagliflozin monotherapy. Furthermore, a UACR reduction exceeding 30% was observed as early as 14 days after initiating combination therapy, meeting the American Diabetes Association recommended threshold for slowing CKD progression (Agarwal et al., 2025). These findings support the use of combination therapy for treating individuals with HFpEF and CKD, indicating that SGLT2is and GLP-1 RAs may have synergistic effects with finerenone. Moreover, a meta-analysis indicated that the utilization of SGLT2is was associated with a reduced risk of severe hyperkalemia and potentially improved adherence to MRAs, consistent across participants with and without baseline MRAs use (Agarwal et al., 2022; Neuen et al., 2022). However, these findings require further confirmation in RCTs with sufficient sample sizes. The STEP-HFpEF series of studies demonstrated that in obese patients with HFpEF, the weekly dose of 2.4 mg semaglutide for 1 year markedly decreased body weight and enhanced HF symptoms, physical activity restrictions, and exercise capacity in comparison to placebo (Kosiborod et al., 2023; Kosiborod et al., 2024). This landmark finding provides a strong foundation for further investigation of GLP-1 RAs in patients with HFpEF who also have obesity and T2DM. Additional clinical trials are assessing the impact of GLP-1 RAs on obese patients with HFpEF. For instance, 700 obese HFpEF patients with or without T2DM will be included in the ongoing SUMMIT research to assess the effectiveness of the GLP-1/GLP-1 R dual agonist tirzepatide (Gallo and Volpe, 2024). Thus, in addition to combination with SGLT2is, finerenone may also be combined with GLP-1 RAs for dual or even triple therapy.

In summary, finerenone demonstrates promising cardiorenal protective potential and safety in patients with HFpEF, particularly those with concomitant CKD or T2DM. Given the current limited treatment options for HFpEF, finerenone offers a therapeutic pathway with a unique mechanism and well-defined target. The ongoing REDEFINE-HF trial will focus on patients hospitalized for acute HFmrEF/HFpEF, representing a more acute patient population compared to previous studies. The CONFIRMATION-HF trial will assess the effectiveness of early combination therapy with finerenone and SGLT2is in patients hospitalized for acute HF, irrespective of LVEF. The FINALITY-HF trial will assess the clinical efficacy and safety of finerenone in HFrEF patients who are intolerant to or ineligible for steroid MRAs (Taub et al., 2025). The conclusive outcomes of these trials are anticipated to yield superior evidence-based justification for the utilization of finerenone in HFpEF patients (Table 2). In the future, exploring synergistic effects with drugs such as SGLT2is and GLP-1 RAs, tailored to specific patient phenotypes, will become a key direction for personalized and precision treatment of HFpEF.

**TABLE 2:** Planned and ongoing clinical trials with MRAs in HF.

Trial (NCT)	Design	MRAs	Estimated enrollment	Trial population	Primary composite outcome	Phase	Anticipated completion data
Finerenone							
REDEFINE-HF (NCT06008197)	Multicenter, randomized, double-blind, placebo-controlled, event-driven phase 3 trial	Finerenone vs. Placebo	5200	Hospitalized HF patients with mildly reduced or preserved EF	Composite total of HF events (first and subsequent HF hospitalizations, urgent visits for worsening HF) and CV death	Recruiting	April 2026
CONFIRMATION-HF (NCT06024746)	Multicenter, randomized, controlled, open-label phase 3 trial	Finerenone plus empagliflozin vs. Standard care	1500	Current hospitalization or recently discharged with the primary diagnosis of HF	Hierarchical composite of the following • Time to all-cause mortality • Number of total HF events • Time to first HF event • Difference of 5 points or greater on the KCCQ-TSS assessed by the win-ratio method	Recruiting	August 2026
FINALITY-HF (NCT06033950)	Multicenter, randomized, double-blind, placebo-controlled phase 3 trial	Finerenone vs. Placebo	2600	Symptomatic HFrEF patients not on sMRA due to history of intolerance, contraindication, or ineligibility for treatment	Time to first CV death or HF event	Recruiting	April 2028
Spironolactone							
SPIRRIT-HFpEF (NCT02901184)	Multicenter, randomized, controlled, open-label phase 3 trial	Spironolactone vs. Standard care	2000	Symptomatic HF patients with elevated NT-proBNP/BNP and EF ≥ 40%	Incidence rate for total HF hospitalizations or CV death	Recruiting	December 2026
Balcinrenone							
Balanced-HF (NCT06307652)	Multicenter, randomized, double-blind, placebo-controlled phase 3 trial	Balcinrenone plus dapagliflozin vs. Dapagliflozin	4800	Symptomatic HF patients with a recent HF event within 6 months (hospitalization or urgent visit) and eGFR ≥ 20 to<60 mL/min/1.73 m2	Time to first occurrence of any of the components of the composite • CV death • HF hospitalisation • HF event without hospitalisation	Recruiting	June 2027

*Abbreviations*: BNP, B-type natriuretic peptide; CV, cardiovascular; EF, ejection fraction; eGFR, glomerular filtration rate; HF, heart failure; HFrEF, heart failure with reduced ejection fraction; KCCQ-TSS, Kansas City Cardiomyopathy Questionnaire-Total Symptom Score; MRAs, mineralocorticoid receptor antagonists; NT-proBNP, N-terminal pro-B-type natriuretic peptide; sMRA, steroidal mineralocorticoid receptor antagonist; Vs., versus.

## Potential cardiorenal protective mechanisms of finerenone

**FIGURE 1:**
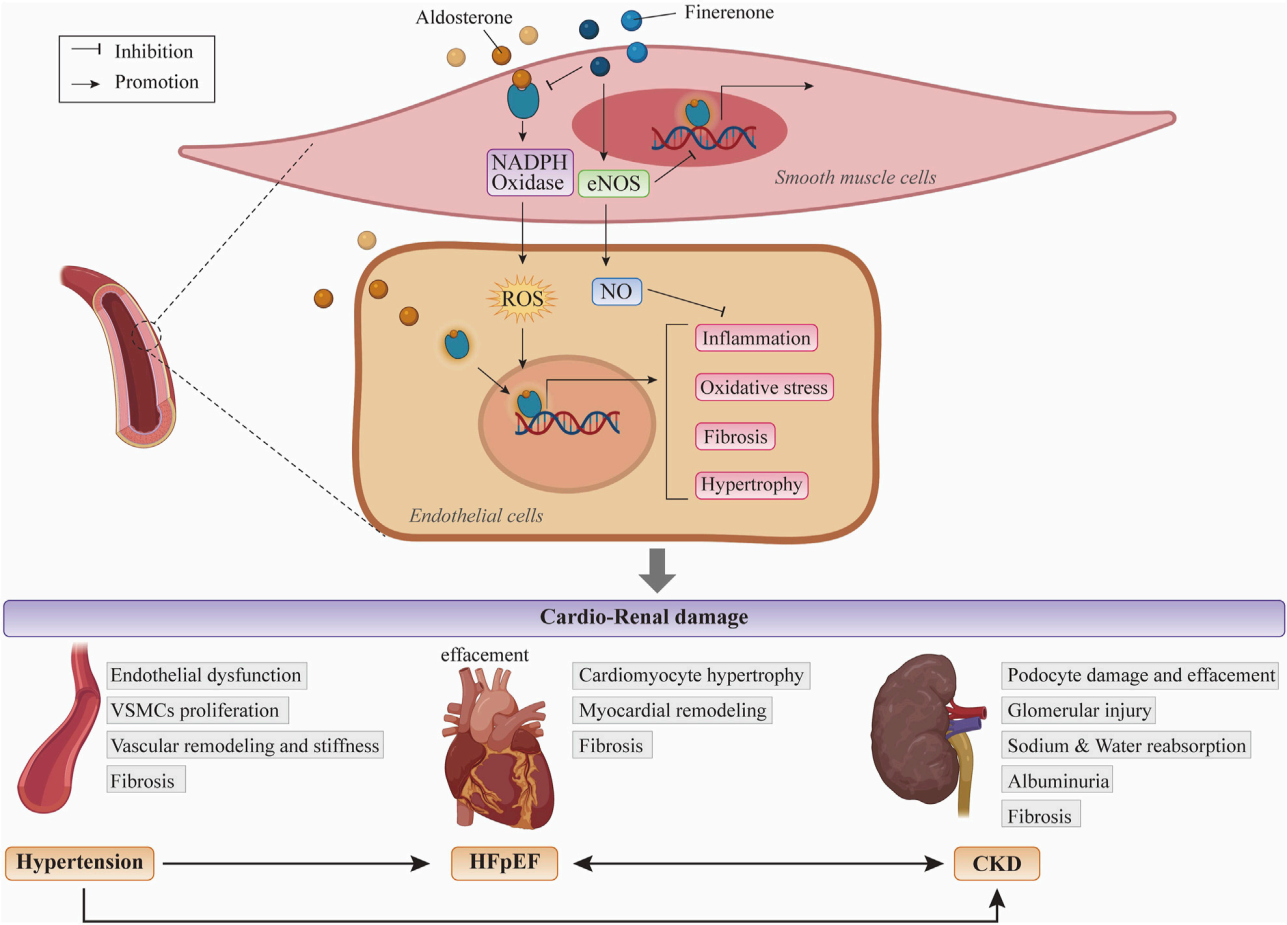
Mechanisms of MR overactivation leading to cardiac and renal injury and the specific effects of finerenone Upon binding to mineralocorticoids such as aldosterone, MR undergoes homodimerization and translocates into the nucleus, where it binds to specific hormone response elements to initiate gene transcription. MR overactivation plays a crucial role in promoting NADPH oxidase activation and enhancing ROS accumulation within VSMCs and ECs, thereby inducing oxidative stress. Mineralocorticoids cause ECs dysfunction and inflammatory cell infiltration, recruit multiple inflammatory mediators, promote VSMCs proliferation, and lead to vascular fibrosis and reduced compliance. MR hyperactivation also affects the kidneys, exacerbating podocyte injury, podocyte foot process loss, and glomerular damage. In the heart, MR overactivation exacerbates HF, myocardial remodeling, and fibrosis. Finerenone binds to MR, indirectly inhibiting mineralocorticoid binding to MR and its nuclear translocation, thereby blocking a cascade of signaling pathways leading to tissue injury. Abbreviations: ECs: endothelial cells; HF: heart failure; MR: mineralocorticoid receptor; NADPH: nicotinamide adenine dinucleotide phosphate; ROS: reactive oxygen species; VSMCs: vascular smooth muscle cells.

MR overactivation enhances nicotinamide adenine dinucleotide phosphate oxidase activity, increasing ROS accumulation in VSMCs and ECs, thereby triggering a cascade of oxidative stress responses in adult rat models (Sinphitukkul et al., 2019). Furthermore, elevated aldosterone levels induce water-sodium retention and sodium overload, subsequently precipitating inflammatory responses and fibrotic progression. These factors influence the heart, leading to myocardial hypertrophy and ventricular remodeling (Connell and Davies, 2005). In the kidneys, MR activation exacerbates podocyte injury and foot process effacement, glomerular hypertrophy, VSMCs proliferation, and ECs dysfunction, resulting in renal vascular remodeling (Tesch and Young, 2017). Consequently, targeted blockade of MR hyperactivation can improve oxidative stress, inflammation, and fibrosis mediated by this pathway (Figure 1). Owing to its equitable distribution in the heart and kidneys, finerenone offers cardiorenal protective benefits. The non-steroidal structure of finerenone enables a unique binding mechanism, exhibiting high selectivity for the MR. In comparison to spironolactone, finerenone binds to MR as an unstable ligand-receptor complex, prevents cofactor recruitment to MR, and functions as an inverse agonist to regulate downstream pro-inflammatory and pro-fibrotic gene expression profiles independently of aldosterone (Agarwal et al., 2021; Barrera-Chimal et al., 2018; Buonafine et al., 2018; Grune et al., 2018). Finerenone is more efficient than the steroidal MRA eplerenone at inhibiting MR cofactor binding in the presence of aldosterone, as steroidal MRAs operate as partial agonists for aldosterone cofactor recruitment (Kolkhof and Borden, 2012).

Finerenone has been evaluated in multiple rodent models related to HFpEF. Several preclinical investigations have shown that finerenone exhibits significant anti-inflammatory and anti-fibrotic effects (Grune et al., 2018; Kolkhof et al., 2014; Lima-Posada et al., 2023). Furthermore, it markedly reduces myocardial hypertrophy, lowers plasma NT-proBNP levels, and improves parameters associated with early signs of diastolic and systolic dysfunction (Kolkhof et al., 2014). Studies indicate that finerenone reduces macrophage accumulation in the heart within models of cardiac dysfunction induced by short-term isoproterenol administration (Grune et al., 2018). Across deoxycorticosterone acetate (DOCA)-salt plus uninephrectomy mouse and rat models, a short-term isoproterenol-induced myocardial injury mouse model, and Zucker fa/fa rat models, finerenone has exhibited significant anti-fibrotic properties in the heart (Lima-Posada et al., 2023; Lachaux et al., 2018; Luettges et al., 2022). These antifibrotic effects were mediated by finerenone-induced MR cofactor binding inhibition, thereby suppressing the gene expression of the pro-fibrotic protein tenascin-X (TNX). TNX is an extracellular matrix glycoprotein playing a key role in tissue remodeling, inflammatory responses, myocardial injury, and fibrosis. Compared to eplerenone, finerenone treatment significantly reduced TNX expression in mice (Grune et al., 2018; Matsumoto and Aoki, 2020). Lavall et al. (Lavall et al., 2019) demonstrated in neonatal rat cardiac fibroblasts that finerenone markedly suppressed aldosterone-induced upregulation of connective tissue growth factor and lysyl oxidase--both known promoters of fibrotic extracellular matrix formation. Invasive hemodynamic monitoring demonstrated that finerenone reduced LV end-diastolic pressure across multiple HF animal models and improved the end-diastolic pressure-volume relationship (Pieronne-Deperrois et al., 2021). Research indicates that individuals with HFpEF demonstrate early impairment of LV longitudinal systolic function, primarily related to dysfunction of subendocardial longitudinal myocardial fibers, which can be assessed by speckle-tracking echocardiography using global longitudinal strain (GLS) (Collier et al., 2017; Pieske et al., 2020). Research demonstrates that finerenone significantly improves GLS in multiple HFpEF mouse models, suggesting its protective effect on early systolic function (Luettges et al., 2022). Furthermore, in transgenic mice with cardiac-specific Rac1 overexpression, continuous 5-month treatment with finerenone significantly suppressed enlargement of both end-diastolic and end-systolic LV volumes, suggesting its potential to prevent Rac1-activated ventricular dilated remodeling (Lavall et al., 2019). Notably, across different rodent models, finerenone demonstrated superior efficacy compared to eplerenone at equivalent sodium-excreting diuretic doses in reducing myocardial hypertrophy, cardiac fibrosis, and improving LV function (Agarwal et al., 2021; Grune et al., 2018; Grune et al., 2016).

Finerenone selectively inhibits MR, hence obstructing pro-inflammatory signaling pathways in CKD and reducing the synthesis and secretion of pro-inflammatory mediators including IL-4 and TNF-α (Barrera-Chimal et al., 2018; Komada and Muruve, 2019). The use of finerenone into a triple treatment regimen comprising RAAS, SGLT-2, and MR inhibitors demonstrates potential in markedly postponing CKD development and mitigating residual interstitial inflammation (Zhu et al., 2023). Furthermore, finerenone diminishes the development of impaired vascular endothelium by inhibiting VSMCs proliferation and decreasing ECs apoptosis. Dutzmann et al. (2017) systematically investigated finerenone’s effects *in vitro* cell cultures and mouse models of vascular injury. They found that finerenone dose-dependently suppressed aldosterone’s promotion of VSMCs proliferation. In cultured ECs, finerenone also significantly suppressed aldosterone-induced apoptosis, suggesting a protective effect in vascular remodeling regulation. Kolkhof et al. (2014) found that in rat CKD models, compared to an equivalent dose of eplerenone, finerenone treatment significantly downregulated gene expression associated with glomerular hypertrophy, proteinuria, and renal inflammation. In mouse models of renal fibrosis induced by ureteral obstruction and ischemia-reperfusion injury, finerenone exhibited marked direct anti-fibrotic effects by reducing myofibroblast numbers and collagen deposition, suggesting its ability to inhibit renal interstitial remodeling independently of hemodynamic mechanisms (Kolkhof et al., 2014; Droebner et al., 2021). Gil-Ortega et al. (2020) conducted studies using MWF rats (a naturally occurring CKD model with mesenteric arterial stiffness) and found that compared to controls, finerenone significantly reduced arterial stiffness, decreased plasma expression of matrix remodeling proteins such as matrix metalloproteinase-2 (MMP-2) and MMP-4, and improved oxidative stress status, including reduced superoxide anion levels and increased nitric oxide production.

Preclinical investigations indicate that finerenone provides cardiorenal protection with negligible effects on serum potassium levels, implying diminished side effects for this innovative non-steroidal MRAs (Orena et al., 2013). For example, in animal models of rapidly progressive glomerulonephritis, the finerenone precursor BR-4628 significantly suppressed renal injury through anti-inflammatory and anti-fibrotic actions without affecting urinary sodium and potassium excretion or inducing hyperkalemia (Ma et al., 2015). The observed favorable benefits occurred even at finerenone doses that did not elicit noticeable hemodynamic changes, indicating that finerenone may safeguard the heart and kidneys independently of blood pressure (Kolkhof et al., 2021).

## Future direction

Evidence for HFpEF treatment remains overall very limited, primarily owing to its complex pathophysiology and markedly variable phenotype. MR represents a well-defined target for HF therapy, and the evidence trail for the novel non-steroidal MRA finerenone continues to strengthen. From Phase II to Phase III clinical researches, from RCTs to meta-analysis, and from DKD patients to the broader HF population, mounting evidence supports finerenone’s clear HF benefit. The favorable outcomes from the FINEARTS-HF study signify a significant advancement in validating the efficacy and safety of finerenone for treating individuals with HFmrEF/HFpEF. This advancement will help optimize future HFpEF treatment strategies. To further determine finerenone’s potential application along the whole LVEF range, a series of trials are currently underway, including REDEFINE-HF, CONFIRMATION-HF, and FINALITY-HF. These researches aim to broaden finerenone’s application across diverse HF phenotypes and improve outcome through novel management strategies, including early use of non-steroidal MRAs combined with SGLT2is. However, HFpEF’s high heterogeneity, stemming from multiple pathophysiological processes, poses challenges for improving outcome through universal definitions and standardized drug therapies. The emerging concept of phenotyping aims to further classify individuals with relevant comorbidities and mutually exclusive pathophysiologies, laying the groundwork for exploring distinct risk profiles, clinical trajectories, and drug responses among HFpEF patients (Peters et al., 2023; Shah et al., 2015). Furthermore, optimizing combination therapy with existing medications for patients with multiple comorbidities represents an urgent research priority in HFpEF. For instance, finerenone combined with SGLT2is may be considered for patients with concurrent CKD and T2DM; for patients with hypertension and T2DM, angiotensin receptor neprilysin inhibitors (ARNIs) combined with SGLT2is may be considered; for patients with obesity and T2DM, GLP-1 RAs combined with SGLT2is could be taken into consideration (Gevaert et al., 2022; Zawadzka et al., 2022). Regrettably, the majority of existing information is empirical, requiring future RCTs to elucidate appropriate management options for various comorbidities. In the near future, as the pathophysiological heterogeneity of HFpEF becomes better elucidated, identifying patient subgroups responsive to finerenone based on clinical characteristics, biomarkers, and imaging phenotypes will be a key direction for enhancing clinical efficacy.

## Conclusion

The pathogenesis of HFpEF is complex and its phenotype exhibits high heterogeneity, with limited evidence currently available for effective treatment. However, during the preceding 6 decades, MRAs have evolved from potassium-sparing diuretics into novel potent nonsteroidal agents, offering promising prospects for HFpEF management. Recent large-scale clinical researches, including FIDELIO-DKD, FIGARO-DKD, and FINEARTS-HF, have demonstrated that finerenone significantly improves cardiac and renal outcome in patients while exhibiting favorable safety and tolerability profiles. Beyond these clinical findings, preclinical studies indicate that finerenone modulates multiple pathophysiological parameters associated with HFpEF. Future research should further explore the synergistic effects of finerenone with SGLT2is and GLP-1 RAs to develop personalized, precision therapeutic strategies for HFpEF patients with different phenotypes. As clinical evidence mounts, finerenone is set to assume a pivotal position in the holistic care plan for HFpEF.

## Glossary

ACEIs: angiotensin-converting enzyme inhibitors
ARBs: angiotensin II receptor blockers
ARNIs: angiotensin receptor neprilysin inhibitors
BNP: B-type natriuretic peptide
BP: blood pressure
CI: confidence interval
CKD: chronic kidney disease
CKM: cardiovascular-kidney-metabolic
CV: cardiovascular
DKD: diabetic kidney disease
DOCA: deoxycorticosterone acetate
ECs: endothelial cells
EF: ejection fraction
eGFR: estimated glomerular filtration rate
GLP-1 RAs: glucagon-like peptide-1 receptor agonists
GLS: global longitudinal strain
HF: heart failure
HFmrEF: heart failure with mildly reduced ejection fraction
HFpEF: heart failure with preserved ejection fraction
HFrEF: heart failure with reduced ejection fraction
HR: hazard ratio
IL-6: interleukin-6
KCCQ: Kansas City Cardiomyopathy Questionnaire
LVEF: left ventricular ejection fraction
MCP-1: monocyte chemoattractant protein-1
MMP-2: matrix metalloproteinase-2
MMP-9: matrix metalloproteinase-9
MR: mineralocorticoid receptor
MRAs: mineralocorticoid receptor antagonists
NA: not available
NT-proBNP: N-terminal pro B-type natriuretic peptide
NYHA: New York Heart Association
P: probability
RAAS: renin-angiotensin-aldosterone system
RASIs: renin-angiotensin system inhibitors
RCTs: randomized controlled trials
ROS: reactive oxygen species
SGLT2is: sodium-glucose cotransporter-2 inhibitors
T2DM: type 2 diabetes mellitus
TGF-β: transforming growth factor-beta
TNF-α: tumor necrosis factor-alpha
TNX: tenascin-X
UACR: urinary albumin-to-creatinine ratio
Vs.: versus
VSMCs: vascular smooth muscle cells
